# Wavelet-Enhanced CNN for Breast Ultrasound Classification Under Speckle Noise

**DOI:** 10.3390/biomedicines14051151

**Published:** 2026-05-19

**Authors:** Ratapong Onjun, Tanakorn Sritarapipat, Sayan Kaennakham

**Affiliations:** 1 Integrated Science and Innovation Program, Institute of Science, Suranaree University of Technology, 111 University Avenue, Muang, Nakhon Ratchasima 30000, Thailand; d6400798@g.sut.ac.th; 2 School of Mathematics and Geoinformatics, Institute of Science, Suranaree University of Technology, 111 University Avenue, Muang, Nakhon Ratchasima 30000, Thailand; tanakorn.s@sut.ac.th

**Keywords:** breast ultrasound classification, wavelet pooling, speckle noise robustness, symlet wavelets, medical image analysis

## Abstract

**Background/Objectives:** Ultrasound is widely used for breast cancer screening and diagnosis, particularly in low- and middle-income settings, but its diagnostic reliability is often compromised by speckle noise that degrades lesion margins and tissue texture. This study proposes a compact convolutional neural network architecture that replaces standard max or average pooling layers with wavelet-based pooling using Symlet families, and optionally includes wavelet-domain preprocessing to suppress input noise. **Methods:** We conducted 108 experiments across six pooling configurations (avg, max, Sym2 ± preprocessing, Sym4 + preprocessing, Sym6 + preprocessing), two network depths, three batch sizes, and three simulated speckle levels (0%, 10%, 20%). **Results:** The proposed wavelet-based pooling framework showed consistently stronger in-domain performance than conventional pooling strategies across clean and speckle-corrupted settings, with the Sym2 + preprocessing configuration giving the best overall results. The model achieved 93.90% accuracy and 98.89% ROC AUC under clean internal test conditions and maintained stable performance under increased simulated noise levels. However, external validation on the independent BrEaST-Lesions-USG dataset revealed substantial performance degradation, with accuracy decreasing to 53.97% and ROC AUC to 0.4713, indicating limited cross-dataset generalization. **Conclusions:** These findings suggest that wavelet pooling is an effective architectural modification for improving in-domain robustness under controlled perturbation, although additional strategies are still required before reliable real-world deployment can be claimed.

## 1. Introduction

Breast cancer remains one of the most pervasive and deadly cancers among women worldwide. The burden is especially pronounced in low- and middle-income countries (LMICs), where the lack of advanced imaging capabilities and limited resources hinder early detection and treatment, exacerbating mortality and healthcare costs [1]. In this context, affordable and accessible diagnostic tools are imperative. Among imaging modalities such as mammography, MRI, and CT, ultrasound stands out due to its non-ionizing nature, portability, cost-efficiency, and suitability for young women with dense breast tissue, conditions where mammography often underperforms [2,3]. Furthermore, ultrasound is routinely employed to differentiate cystic from solid masses and to guide biopsies, a role increasingly reinforced by recent hardware–software co-design advances [4]. However, ultrasound is bilaterally challenged by speckle noise, a granular, multiplicative interference that impairs image clarity, compromises boundary delineation, and degrades contrast, complicating both manual and automated interpretation [5,6].

Moreover, ultrasound images exhibit substantial variability in lesion appearance, including echogenicity, shape, and margin definition, further complicating diagnosis [7]. Traditional diagnostic frameworks, often rule-based, fail to generalize across this heterogeneity. In response, deep learning, especially convolutional neural networks (CNNs), has shown extraordinary promise in analyzing medical imagery by autonomously extracting hierarchical features [8].

Over the last decade, convolutional neural networks (CNNs) have become foundational in computer vision and medical image analysis due to their hierarchical feature extraction capability, which enables them to automatically learn low- to high-level patterns directly from imaging data. They have demonstrated superior performance in a variety of medical applications, including mammography, histopathology, and CT scans, often surpassing traditional image processing and handcrafted feature techniques. Convolutional Neural Networks (CNNs) have revolutionized medical image analysis by automating feature extraction and enhancing diagnostic accuracy. However, when applied to ultrasound-based breast cancer detection, a prominent issue is their vulnerability to noise [9]. However, when applied to ultrasound-based breast cancer detection, CNNs still face critical challenges that hinder their generalizability in real-world scenarios. A prominent issue is their vulnerability to noise. CNNs trained on curated, noise-free datasets often exhibit drastic performance drops when evaluated on images containing real-world degradation such as speckle noise. This vulnerability is particularly concerning in LMIC settings, where lower-end imaging equipment, operator skill variability, and inconsistent acquisition protocols contribute to lower-quality scans. Speckle noise, in particular, manifests as random granular interference that distorts lesion texture and boundaries, key features required for accurate classification, making standard CNNs prone to misclassification in such conditions [10].

Another limitation lies in the design of pooling layers, which are fundamental to reducing spatial dimensions and capturing invariant features. While max pooling and average pooling have long served as standard down-sampling techniques, both exhibit limitations in the context of noisy, high-variability ultrasound images. Max pooling can be overly sensitive to high-intensity outliers or isolated noise spikes, leading to unstable feature extraction. On the other hand, average pooling tends to overly smooth fine details, which can cause loss of diagnostically relevant texture and boundary information. These shortcomings are particularly problematic in breast ultrasound, where subtle cues, such as shadowing patterns or irregular lesion margins, are critical for distinguishing between benign and malignant tumors [11]. Recent studies have explored wavelet-based pooling as an alternative to traditional approaches, where discrete wavelet transforms decompose feature maps into multi-scale frequency components, potentially preserving both spatial structure and frequency information critical for medical image analysis. In breast ultrasound classification, wavelet pooling has demonstrated superior performance over conventional pooling methods, achieving higher accuracy rates while maintaining structural integrity of diagnostically relevant features [12]. Furthermore, traditional pooling layers are not inherently designed to consider structural integrity under distortion, nor do they incorporate multiscale or frequency-aware information, which could otherwise help to retain signal fidelity. As a result, these limitations collectively diminish the effectiveness of standard CNN architectures in ultrasound-based diagnosis, especially in the presence of complex, noise-contaminated inputs.

Despite the widespread adoption of convolutional neural networks in medical imaging, there remains a conspicuous gap in the literature regarding their adaptation to noisy ultrasound environments, particularly for breast cancer detection. Most existing studies tend to assume ideal input conditions, training and testing models on clean, high-quality images while largely overlooking the effects of real-world imaging degradation, such as speckle noise, which is inherent in ultrasound acquisition. As a result, many reported accuracies are not reflective of clinical performance. Furthermore, pooling operations, a critical component in CNNs that controls spatial resolution and abstraction, are rarely scrutinized beyond the use of traditional max or average pooling. The potential of alternative, structure-preserving pooling strategies remains significantly underexplored. While wavelet transforms, especially those based on the Symlet family, are well established in classical signal processing for their multiscale, shift-invariant, and frequency-localized properties, their integration into deep learning frameworks, particularly as learnable or fixed pooling operators, has been minimal [13,14].

Previous research has demonstrated that wavelet pooling significantly outperforms traditional pooling methods for histopathological breast cancer image analysis while maintaining structural integrity and reducing overfitting issues. The wavelet transform-based pooling approach effectively decomposes feature maps into multiscale frequency components, preserving both spatial and textural information critical for accurate lesion classification [15]. These transforms offer a compelling advantage by simultaneously preserving both spatial structure and frequency information, making them a promising solution for the kinds of texture-rich and noise-prone data typically seen in medical ultrasound. In particular, Symlet wavelets, with their nearly symmetrical design and compact support, are highly suitable for capturing boundary features and reducing aliasing effects under distortion [16].

To address these limitations, this study proposes a novel CNN architecture that leverages wavelet-based pooling, specifically Symlet transforms, as a replacement for conventional pooling operations. The proposed approach is further enhanced by the optional inclusion of a preprocessing pipeline that applies wavelet-domain denoising to the input images prior to feature extraction. This dual-stage enhancement, targeting both input-level noise and internal representation robustness, is systematically evaluated across various Symlet orders (Sym2, Sym4, Sym6). In this way, the study not only tests the efficacy of different wavelet scales but also investigates how these configurations interact with network depth and batch size. The framework is designed to simulate realistic clinical conditions, including varying noise intensities, and is benchmarked against a standard architecture (e.g., VGG16) using multiple performance metrics. This comprehensive design allows for a quantitative and statistically rigorous assessment of how wavelet pooling influences learning under non-ideal conditions, thereby contributing a novel and practically relevant solution to the challenge of robust breast ultrasound classification.

The contributions of this study can be summarized as follows. First, we introduce a breast ultrasound classification framework in which conventional pooling operations are systematically replaced by Symlet-based wavelet pooling, with and without wavelet-domain preprocessing at the input stage. Second, we provide a structured comparison across multiple pooling strategies, network depths, batch sizes, and controlled speckle perturbation levels, allowing a more comprehensive view of architectural robustness than a single-setting benchmark. Third, we complement standard classification metrics with statistical significance analysis and Grad-CAM visualization in order to examine not only predictive accuracy but also stability and lesion-focused feature behavior. Finally, by including external validation on an independent dataset, we show that strong in-domain performance does not necessarily imply successful cross-dataset transfer, thereby clarifying both the strengths and the present limitations of the proposed design.

## 2. Related Work

Early studies applying deep learning to breast ultrasound (BUS) focused on demonstrating that convolutional neural networks (CNNs) could improve on hand-crafted features and conventional pipelines. The release of curated datasets such as BUSI helped to standardize evaluation and made it easier to compare models for benign–malignant classification and segmentation under similar settings [17]. With these resources, CNN-based systems began to show consistent gains for lesion detection, localization, and diagnosis, often outperforming older texture descriptors and rule-based approaches [18,19,20,21]. Multicenter work then moved the field toward more realistic testing, showing that models trained with careful protocols can generalize across scanners and hospitals, which is essential if systems are to be trusted in routine practice [22]. Several reviews now summarize this progress. In short, the literature shows that CNNs can work well on BUS, but it also highlights the need to evaluate models more rigorously when image quality is imperfect, especially in settings where hardware, acquisition, and operator factors are harder to control [23,24,25].

A major reason ultrasound is challenging is speckle noise, which is signal-dependent and multiplicative. Classical despeckling methods try to strike a balance between smoothing noise and preserving structure. Examples include speckle-reducing anisotropic diffusion (SRAD) and non-local means filters adapted for ultrasound statistics, along with families of adaptive filters that target different spatial scales [26,27]. Surveys comparing compounding, diffusion, bilateral, and patch-based methods generally agree that these tools can improve visual quality, but they also warn that aggressive filtering may blur lesion boundaries or dampen subtle texture cues that matter for diagnosis [28]. With deep learning, the community has moved toward learned priors and multi-task designs that combine denoising with other objectives (e.g., resolution enhancement), aiming to suppress speckle without erasing useful details [29]. Unsupervised and self-supervised approaches are especially attractive because obtaining “clean” ground truth is not realistic in vivo; recent “speckle-to-speckle” frameworks learn from pairs of differently speckled acquisitions or simulated independent speckle fields and report steady noise reduction with better downstream behavior [30,31]. However, in most reports denoising is treated as a separate pre- or post-processing step. Few studies look closely at how noise suppression should interact with a classifier’s internal representation, particularly the down-sampling stages where information is routinely discarded and where good design could make the network itself more robust.

This is where wavelet-based ideas become relevant. Wavelet CNNs enrich feature extraction by adding discrete wavelet transforms (DWT) so that the network sees information in both spatial and spectral domains. Architectures such as MWCNN replace strided down-sampling with DWT/IWT to reduce aliasing, preserve edges, and better capture multiscale patterns, benefits that have translated well in restoration tasks and increasingly in recognition [32,33,34]. In ultrasound-based breast cancer detection, wavelet-enhanced CNNs have demonstrated superior classification performance, with wavelet convolutional neural networks (WCNNs) outperforming traditional deep learning models such as ResNet50 and MobileNetV2 [35]. A related line of work proposes wavelet pooling as a drop-in replacement for max/average pooling. Instead of taking a local max or mean, the network decomposes a feature map into sub bands and typically retains, fuses, or learns from the approximation coefficients, which helps keep structure that would otherwise be lost [33,34,36]. Recent comparative studies on diabetic retinopathy classification have demonstrated that Symlet wavelets outperform Haar and Daubechies-2 variants, particularly excelling at smaller batch sizes. This evidence further supports the potential of wavelet pooling variants in medical imaging applications where texture preservation and noise resilience are critical [37]. Similarly, wavelet convolutional neural networks (WCNNs) integrating wavelet transforms have demonstrated superior performance over standard CNNs in breast cancer detection from histopathological images. The wavelet transform’s ability to decompose signals into low and high-frequency components provides effective feature extraction capabilities that complement hierarchical CNN learning [38]. Multiple-wavelet and learnable-wavelet variants push this idea further, showing faster convergence and competitive accuracy against standard pooling on natural image benchmarks [34,36]. While much of this evidence comes from outside ultrasound, the signal processing rationale maps well to BUS: coherent imaging generates speckle that is strongest in high-frequency detail, and DWT-based operators can separate scale and orientation in ways a 2D max/mean cannot. What remains underexplored is a systematic evaluation of wavelet pooling for BUS classification (not only segmentation) under controlled noise levels, precisely the gap addressed in the present study.

Beyond wavelets, several alternatives to standard pooling have been tested to reduce information loss and improve invariance. Stochastic pooling regularizes selection by sampling from activations, fractional max-pooling introduces flexible pooling geometry, generalized-mean (GeM) pooling interpolates between average and max with a trainable exponent, and anti-aliased (blur) pooling inserts proper low-pass filtering before subsampling to improve shift-stability [39,40,41,42]. These methods share a common goal—down-sampling while keeping features stable—but they differ in how they treat local structure and frequency content. In medical imaging, and BUS in particular, the stakes are high because subtle edges (spiculations, echogenic rims) and fine textures can carry diagnostic meaning; if down-sampling blurs them, the classifier may miss important cues. Despite the rich toolbox, relatively few BUS studies have benchmarked multiple pooling operators head-to-head, under explicit noise stress (e.g., controlled speckle levels), and with consistent training/evaluation protocols. That gap motivates architectures that bring wavelet-based pooling into the core of the network and experiments that deliberately vary noise while holding everything else constant so that conclusions are clear.

Finally, evaluation practice matters. Reporting a single accuracy or AUC rarely tells the full story when comparing multiple designs over repeated runs. The machine learning methodology literature recommends non-parametric tests—for example, Friedman rankings with appropriate post hoc procedures (Nemenyi or Holm) for many-model comparisons across datasets or folds, and Wilcoxon signed-rank for paired contrasts—together with visual summaries like critical-difference diagrams to make the evidence easy to interpret [43,44]. When two ROC curves are compared on the same cases, DeLong’s method provides a principled test for correlated AUCs and is widely used in medical imaging [45]. These tools help to separate real improvements from sampling noise, which is crucial when reported differences are a few percentage points and when clinical decisions depend on the model’s reliability. In the context of BUS, combining noise-aware experiments with formal significance testing provides stronger, more reproducible evidence than point estimates alone. This is the motivation behind our study design: we evaluate Symlet-based wavelet pooling (with and without preprocessing) against conventional pooling across defined speckle regimes, we include a well-known CNN baseline for context, and we report results with the statistical procedures recommended in the literature so that performance gains can be interpreted with confidence.

## 3. Materials and Methods

This section details the dataset, controlled noise simulation, optional preprocessing, the proposed CNN architecture and pooling modules, training and benchmarking protocols, evaluation metrics, and statistical analysis. The design choices follow a simple rule: every component should help the model to remain accurate and stable when images are noisy, as is often the case in real clinics.

### 3.1. Dataset and Partitions

We used a curated set of B-mode breast ultrasound images annotated by experts as benign or malignant. To ensure consistent input size, each image was center-padded to preserve aspect ratio (if needed) and resized to 224 × 224 pixels, then normalized to pixel values in the range 0 to 1 by dividing by 255. We applied a stratified split into training/validation/test sets with a ratio of 70/15/15 so that the class balance is similar in each split. Unless stated otherwise, all ablations and baselines use the same splits to keep comparisons fair.

We used the Ultrasound Breast Images for Breast Cancer dataset (Kaggle) [46] for training and internal validation, while the BrEaST-Lesions-USG dataset from The Cancer Imaging Archive (TCIA) [47] served as an independent external validation cohort to assess cross-dataset generalization (Table 1). Because the Kaggle source dataset [46] is distributed as a compiled image collection without patient-level identifiers, a strict patient-level split could not be applied to the internal data. Image-level splitting can therefore allow residual correlation between train, validation, and test partitions whenever multiple frames originate from the same lesion or patient, and this may inflate internal accuracy estimates relative to a true patient-disjoint protocol. To partially mitigate this limitation, the Breast-Lesions-USG dataset [47], which does provide case-level metadata, was used as an entirely independent external cohort (Section 4.6); because the external cohort is disjoint from the training source at the dataset, institution, and patient levels, it constitutes a stricter generalization test than any internal patient-level split would provide. The internal metrics in Section 4.1, Section 4.2, Section 4.3 and Section 4.4 should therefore be read as upper-bound source-domain estimates, while Section 4.6 reports the unbiased cross-cohort performance.

To ensure fair comparison, all baseline and proposed models were trained and evaluated under the same experimental pipeline, including the same train–validation–test partitioning strategy, augmentation procedure, optimizer settings, early-stopping policy, and metric computation scheme. In this way, observed differences can be attributed mainly to the architectural variations, especially the choice of pooling mechanism and preprocessing configuration, rather than to inconsistencies in training protocol.

Data partitioning was performed in a stratified manner at the image level. We acknowledge that patient-level separation would provide a stricter assessment in medical imaging tasks, since image-level splitting may allow residual correlation between subsets when multiple images originate from related clinical sources. This should therefore be considered when interpreting the strong internal performance results.

### 3.2. Controlled Speckle Noise Simulation

Justification of the noise model. Fully developed speckle in coherent ultrasound imaging arises when many sub-resolution scatterers within a resolution cell contribute randomly phased echoes classical results show that the amplitude envelope then follows a Rayleigh distribution, and that speckle is fundamentally signal-dependent and multiplicative rather than additive. After envelope detection, log-compression, and vendor-specific post-processing, the exact first-order statistics deviate from pure Rayleigh and are better described by more general families [48]. For the purposes of CNN robustness testing, however, the property that matters most is not the precise distributional form but the signal-dependent multiplicative structure brighter pixels experience proportionally stronger perturbation because this is what shifts the feature-level input statistics that a classifier must tolerate. A first-order multiplicative Gaussian model of the form

(1) $$I_{\mathrm{noisy}}\;=\;I(1+n),\;n\sim Ν(0,\sigma^{2}),$$

with  σ∈{0.1, 0.2} to parametrize “10%” and “20%” perturbation, preserves this multiplicative signal dependence while remaining computationally simple, fully reproducible, and widely adopted in the ultrasound deep-learning literature [5,10,29]. The Gaussian choice is further supported by the fact that, under moderate-to-high SNR, the Rayleigh envelope becomes approximately Gaussian around its local mean, so a multiplicative Gaussian captures the dominant first-order behavior that a learned feature extractor is sensitive to.

Alignment with real speckle and limitations. We explicitly acknowledge three simplifications that this model does not capture. First, our noise is sampled i.i.d. per pixel, whereas real speckle has a spatial autocorrelation determined by the system point-spread function and typical speckle-cell sizes on the order of the resolution cell, spatially correlated noise would produce slightly different high-frequency content and therefore slightly different interaction with wavelet pooling, though the direction of the effect (higher-order Symlets benefiting more from denoising, see Section 3.5) is expected to be preserved. Second, log-compression and vendor-proprietary speckle-reduction filters present on clinical scanners are not modeled; this means our “20%” perturbation should not be interpreted as a calibrated physical SNR but as an engineering parameter for a stress-test gradient. Third, the Gaussian envelope approximation breaks down in low-SNR regions and is strictly inferior to a Rayleigh envelope sampler. To partially address this last point, an additional analysis comparing Max and Sym2 pooling under Rayleigh-type perturbation is presented in a later section of this manuscript. The qualitative ordering (wavelet pooling > Max pooling at moderate noise, degradation of both at high noise) is consistent with the multiplicative-Gaussian results, supporting the view that the conclusions drawn here generalize at least to a closer-to-canonical speckle distribution. Extending the stress test to log-compressed Fisher–Tippett, Nakagami, and simulator-based PSF-convolved scatterer models is flagged as future work, together with evaluation on clinical data that inherently contain scanner-specific speckle statistics.

### 3.3. Optional Wavelet-Domain Preprocessing (Input Denoising)

For configurations that include preprocessing, we applied wavelet-domain denoising to each input image before it enters the network. Using a two-level 2-D discrete wavelet transform (DWT) with a Symlet basis  symk( k∈{2,4,6}), each image is decomposed into approximation/detail subbands $\;\left\{LL,LH,HL,HH\right\}$ at each level. Let *d* denote a detail coefficient and *N* the number of pixels in the corresponding subband. We estimate the noise scale $\;\hat{\sigma }$ from the first-level diagonal ($\;HH_{1}$) using the robust MAD estimator and apply soft thresholding

(2) $$\tilde{d}\;=\;\mathrm{sign}(d)\max(|d|-\lambda ,0),\;\;\lambda \;=\;\hat{\sigma }\sqrt{2\log N}.$$

The denoised image is obtained via the inverse DWT using $\;\left\{LL,\tilde{L}H\;,\tilde{H}L\;,\tilde{H}H\right\}\;$. This step suppresses high-frequency speckle while preserving edges. In our design, preprocessing is disabled for average/max pooling baselines, applied both with and without Sym2 to isolate its effect, and enabled for Sym4/Sym6.

The design of the preprocessing x pooling ablation was asymmetric by construction rather than by omission. We selected Sym2 as the focal candidate because its shorter filter support (4 taps) provides the strongest spatial localization among the Symlet orders we considered (see Section 3.5), which following the Heisenberg-type trade-off that governs time–frequency analysis makes it the most sensitive to fine-scale lesion margins and therefore the most informative configuration in which to isolate the independent effect of input denoising. Running the with/without-preprocessing contrast on Sym2 alone establishes whether preprocessing contributes additional benefit beyond the architectural term if the contrast were instead distributed across Sym2/Sym4/Sym6, the preprocessing signal would be confounded with the Symlet-order signal and the two factors would be less cleanly separable. For Sym4 and Sym6, preprocessing was retained because (i) their longer supports (8 and 12 taps) integrate information over wider spatial windows and therefore benefit more from prior input-level noise suppression, as predicted by the same spatial-selectivity argument, and (ii) both configurations serve as comparators to the Sym2-preprocessed reference rather than as independent probes of the preprocessing effect. We acknowledge that a fully factorial 3 × 2 design would allow the preprocessing × Symlet-order interaction to be characterized, and we explicitly identify this as a limitation the present ablation nevertheless isolates the two effects under Sym2, which is sufficient to support the manuscript’s principal claims.

### 3.4. Proposed CNN Architecture

We use a compact, custom CNN with either 3 or 4 convolutional blocks (denoted B, where $B\in \left\{3,4\right\}$). Each block contains:

1. a 3 × 3 convolution with “same” padding;
2. batch normalization;
3. ReLU activation;
4. one of the pooling layers defined in Section 3.5.

**Figure 1:**
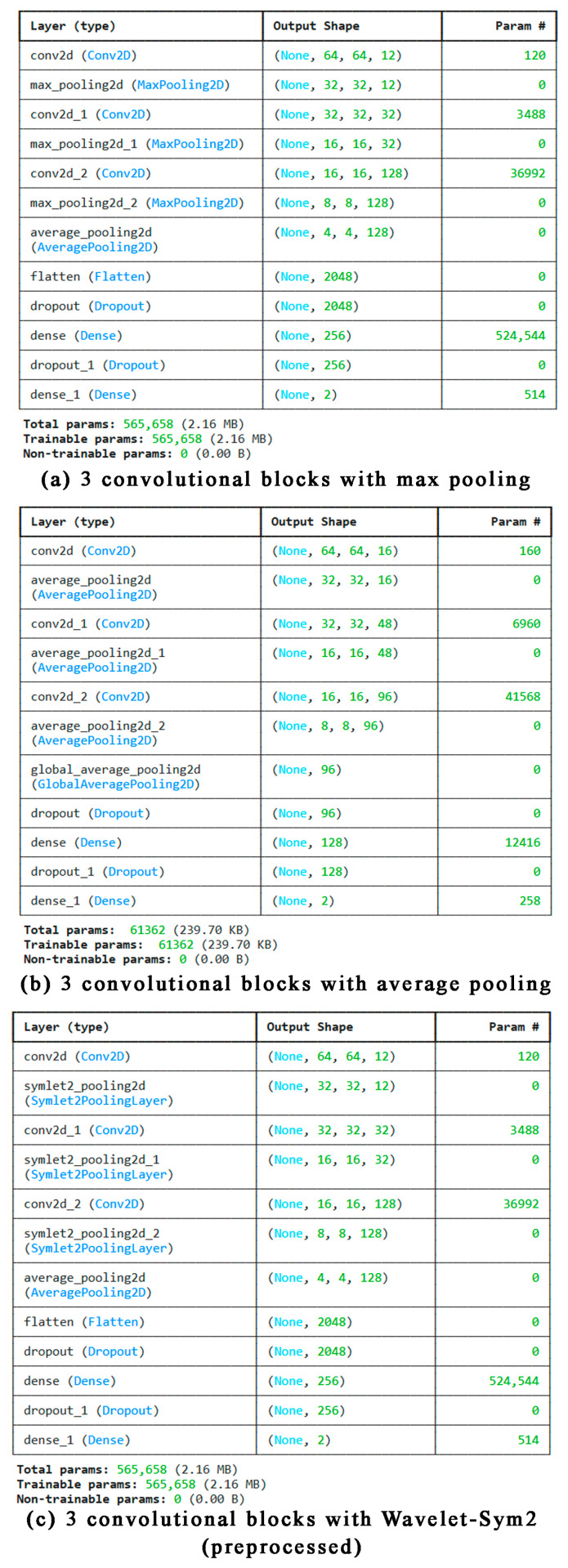
Architectural Diagrams of CNN Models with Different Pooling Strategies.

Channel counts grow with depth: 32→64→128(→256). After the final block, we apply global average pooling (GAP), then a classifier head with a dense layer (128 units, ReLU), dropout *p* = 0.5, and a final dense layer with 2 outputs and softmax. Weights are initialized with He normal initialization. Figure 1 in the manuscript shows schematic diagrams for the max-, average-, and Sym2-pooled variants; the max and wavelet models are parameter-matched so that any gains cannot be attributed to model size, while the average-pooled toy variant is intentionally lighter to illustrate the accuracy–efficiency trade-off.

### 3.5. Pooling Modules

Rationale for Symlet basis selection. The Symlet family was adopted over Haar, Daubechies, Coiflet, and biorthogonal alternatives because Symlets are explicitly constructed as the least-asymmetric orthogonal wavelets with compact support, delivering a near-linear phase response that preserves the spatial location of features across decomposition levels. This phase-preservation property is critical for medical image analysis, because an asymmetric basis would shift lesion boundaries between the approximation and detail subbands, misaligning the pooled feature with the original margin. Haar offers linear phase but only one vanishing moment and a two-tap support, which is too coarse for the texture gradients that characterize benign–malignant differentiation classical Daubechies wavelets share the same vanishing-moment count as Symlets of the same order but exhibit extremal phase, which empirically produces edge displacement artifacts in medical imaging [49]. Coiflet bases are near-symmetric as well but require longer support per vanishing moment, offering no advantage in our setting.

Within the Symlet family, we investigated orders N ∈ {2, 4, 6} to probe the trade-off governed by the filter length (2N taps), the support width (2N − 1), and the number of vanishing moments (N). Sym2 has the shortest support (4 taps, support from 0 to 3) and two vanishing moments, producing sharper spatial localization and stronger response to local intensity transitions the regime in which breast ultrasound lesion boundaries, spiculated margins, and fine echo textural cues reside. Sym4 (8 taps) and Sym6 (12 taps) have progressively longer support and more vanishing moments, which favor sparse representation of smooth regions and polynomials of higher degree but tend to blur narrow edges and blend neighboring structures, especially at deeper layers where a 12-tap filter spans a large fraction of a 14 × 14 feature map. Consistent with the classical Heisenberg-type uncertainty for time–frequency analysis, shorter support trades frequency resolution for spatial precision, and for edge-dominated targets such as BUS lesions this trade favors the lower-order basis. We capped the evaluation at Sym6 because SymN with N ≥ 8 is rarely adopted in the image analysis literature; the support exceeds the receptive field of a single feature-map position in compact networks, and empirical denoising gains plateau or reverse [37]. The experimentally observed ordering Sym2 > Sym4 > Sym6 at clean and low-noise settings is therefore not merely empirical but follows directly from this spatial-vs.-frequency trade-off.

We investigated six pooling configurations:

1. Average pooling, no preprocessing.
2. Max pooling, no preprocessing.
3. Wavelet-Sym2 pooling, no preprocessing.
4. Wavelet-Sym2 pooling, with preprocessing.
5. Wavelet-Sym4 pooling, with preprocessing.
6. Wavelet-Sym6 pooling, with preprocessing.

**Average/Max pooling.** Standard 2 × 22\times 2 windows with stride 2 reduce spatial size by a factor of two.

**Wavelet pooling.** For each feature map $\;F\in {\mathbb{R}}^{H\times W}$, we compute a one-level 2-D DWT with a Symlet basis, obtaining subbands (*LL*, *LH*, *HL*, *HH*). We retain the *LL* subband as the down-sampled feature:

(3) $$P(F)\;=\;LL\;\;=\;\;W_{\mathrm{sym}k}(F){|}_{LL},\;\;k\in \{2,4,6\}.$$

This halves each spatial dimension (like stride-2 pooling) but performs reduction after a learned, multiscale filter bank, which tends to keep structure and push speckle to the discarded high-frequency subbands.

### 3.6. Training Protocol

Loss. We train with the binary cross-entropy loss

(4) $$L_{\mathrm{BCE}}\;=\;-\frac{1}{N}\sum_{i=1}^{N}\sum_{c\in \{0,1\}}y_{i,c}\log{\hat{p}}_{i,c},$$

where $\;{\hat{p}}_{i,c}$ is the predicted probability for class *c* and $\;y_{i,c}\in \{0,1\}$ is one-hot ground truth. To confirm robustness, we also computed the macro-averaged F1 (reported only in evaluation), but the model is optimized with BCE for stability.

**Optimizer and schedule.** We use Adam (learning rate $\;10^{-3},\beta_{1}=0.9,\beta_{2}=0.999),$ $\;L_{2}$ weight decay $\;10^{-4}$, and early stopping on the validation loss with patience of 10 epochs (maximum 100 epochs). A Reduce-on-Plateau scheduler halves the learning rate if validation loss does not improve for 5 epochs.

**Batch size.** We tested $\;\left\{8,16,32\right\}$ for each  B∈{3,4} as part of the ablation in Section 4, because small batches add useful gradient noise when data are heterogeneous.

**Data augmentation.** On-the-fly geometric transforms include random horizontal flips ( p=0.5), rotations $\;\pm 15^{{}^{\circ}}$, translations up to 10% of height/width, and isotropic zoom in [0.9, 1.1]. Intensity jitter is avoided to not distort clinical appearance.

**Reproducibility.** Each configuration is repeated with 5 different random seeds; we report mean  ± standard deviation on the test set.

### 3.7. Baselines and Benchmarking

We compare the proposed model to VMC-NET (a compact baseline with max pooling) and VGG-style transfer learning. For transfer learning, the backbone convolutional layers are initialized from ImageNet and either frozen or partially unfrozen based on validation performance; the classifier head is re-initialized. All baselines use the same input size, splits, augmentation, optimizer, and training budget to keep the comparison controlled.

### 3.8. Evaluation Metrics

We evaluate with standard diagnostic metrics computed from the confusion matrix (*TP*, *FP*, *TN*, *FN*):

(5) $$\mathrm{Accuracy}\;=\;\frac{TP+TN}{TP+FP+TN+FN},\;\;\mathrm{Precision}\;=\;\frac{TP}{TP+FP},$$

(6) $$\mathrm{Recall}\;(\mathrm{Sensitivity})\;=\;\frac{TP}{TP+FN},\;\;\mathrm{Specificity}\;=\;\frac{TN}{TN+FP},$$

(7) $$F1\;=\;\frac{2\cdot \mathrm{Precision}\cdot \mathrm{Recall}}{\mathrm{Precision}+\mathrm{Recall}}.$$

We also compute the area under the ROC curve (AUC) by sweeping the decision threshold and integrating the empirical ROC. Metrics are reported per noise level (0%, 10%, 20%) to make robustness visible. In addition, we record parameter count and single-image inference time (ms) to show practicality.

Calibration metrics. Beyond discrimination, we evaluate the agreement between predicted probabilities and observed positive-class frequencies using two complementary measures. The Brier score quantifies the mean squared difference between each predicted probability and its corresponding binary label, with lower values indicating both better discrimination and better calibration. The Expected Calibration Error (ECE) partitions predictions into ten equal-width confidence bins and computes the weighted average of the absolute difference between the mean predicted confidence and the empirical accuracy within each bin, thereby quantifying how far the model’s stated confidence drifts from its observed correctness. We additionally generate reliability diagrams plotting bin-wise empirical accuracy against bin-wise mean confidence, perfect calibration corresponds to the diagonal. Both metrics are computed on the internal test set and on the Breast-Lesions-USG external cohort to make the calibration shift under domain transfer explicit. Where the analysis indicates miscalibration, we additionally fit Platt scaling and isotonic regression on a held-out portion of the internal validation set and re-evaluate Brier and ECE on the test/external sets; this is reported as a post hoc remediation analysis only and is not used to alter the headline results.

### 3.9. Statistical Analysis

Because we compare multiple pooling strategies across repeated runs and noise conditions, we use non-parametric tests. Let $\;r_{ij}$ be the rank of method *j* on dataset/condition *i* (e.g., 5 random seeds × 3 noise levels). The Friedman statistic is

(8) $$\chi_{F}^{2}\;=\;\frac{12N}{k(k+1)}\left[\sum_{j=1}^{k}{\bar{r}}_{j}^{2}-\frac{k{\left(k+1\right)}^{2}}{4}\right],$$

where *k* is the number of methods and $\;{\bar{r}}_{j}$ is the average rank of method *j* over *N* cases. We reject the null hypothesis that all methods are equivalent if  p<0.05. For selected pairwise comparisons (e.g., best wavelet vs. best conventional pooling at each noise level), we apply the Wilcoxon signed-rank test to paired scores across seeds/conditions. Where relevant (e.g., AUC comparisons between two ROC curves on the same samples), we additionally compute DeLong’s test. Critical-difference (CD) diagrams visualize average ranks and significant cliques. Effect size and uncertainty reporting. Following recommendations for non-parametric reporting in machine learning evaluation [43,50], we complement *p*-values with effect size estimates and 95% confidence intervals so that the magnitude of the observed differences, not only their statistical significance, is documented. For each Wilcoxon signed-rank pairwise contrast we report the matched-pairs rank-biserial correlation $\;r_{rb}$ as defined by Kerby (2014) [51], computed as the difference between the proportions of favorable and unfavorable signed ranks; this statistic is bounded in [−1, 1] and admits the same magnitude conventions as Pearson’s   r(small≥0.10,medium≥0.30,large≥0.50). For the omnibus Friedman test, we report Kendall’s   W as the corresponding effect size, with conventional thresholds of small (0.1), medium (0.3), and large (0.5) [50]. For all reported metrics (accuracy, AUC, F1) we additionally report the 95% confidence interval of the mean across the five random seeds, computed via bias-corrected and accelerated (BCa) bootstrap with 10,000 resamples [52], for AUC differences between paired ROC curves we additionally report DeLong CIs [45] alongside the previously cited DeLong test.

### 3.10. Interpretability (Grad-CAM)

To verify that the network focuses on lesion regions rather than artifacts, we compute Grad-CAM maps from the last convolutional layer. For class cc with score $\;y^{c}$ and feature maps $\;A^{k}$, we calculate importance weights

(9) $$\alpha_{k}^{c}\;=\;\frac{1}{Z}\sum_{i,j}\frac{\partial y^{c}}{\partial A_{ij}^{k}},$$

And the class-discriminative heatmap

(10) $$L_{\mathrm{Grad-CAM}}^{c}\;=\;\mathrm{ReLU}\left(\sum_{k}\alpha_{k}^{c}A^{k}\right).$$

**Figure 2:**
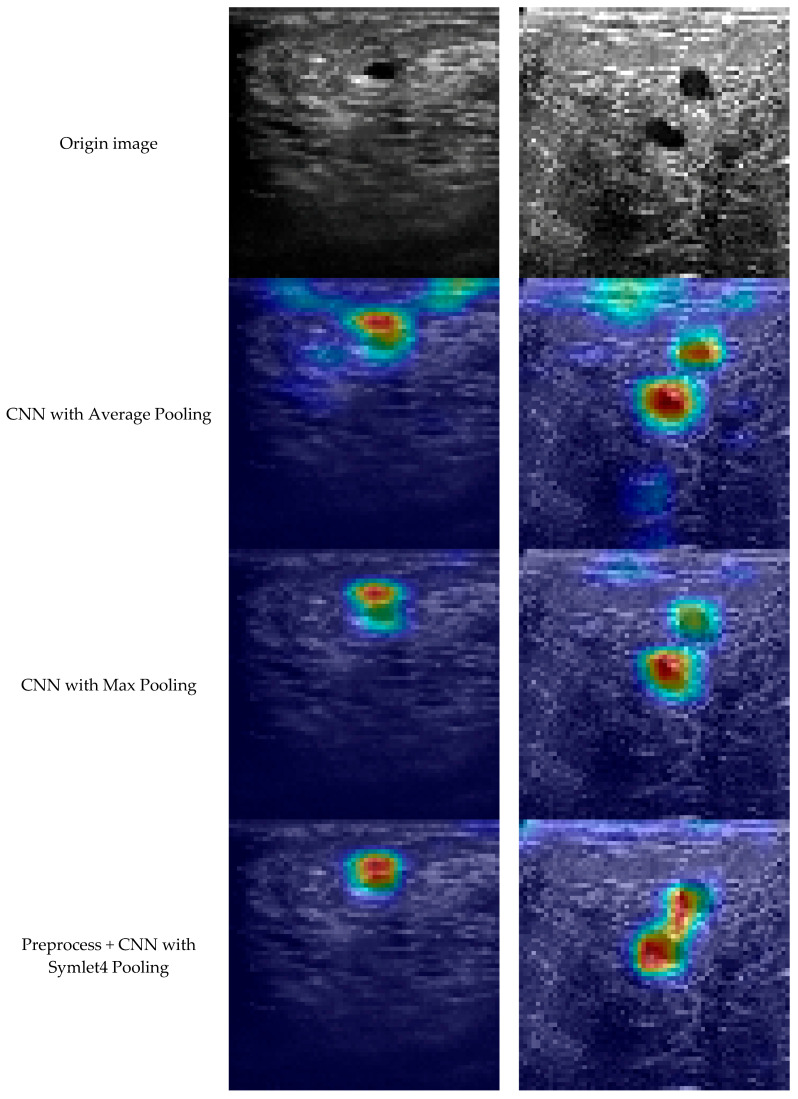
Grad-CAM Heatmaps Showing Discriminative Focus of the CNN Across Different Pooling Methods.

We show qualitative examples in Figure 2 to compare average, max, and wavelet pooling. In our results, wavelet pooling concentrates activation on the mass boundary and core even under noise, which aligns with the quantitative gains.

Prior work has considered metrics such as Attention-on-Lesion (AoL) and Intersection-over-Union (IoU) for evaluating localization quality; however, in this study we focus on the Pointing Game accuracy [53,54].

### 3.11. Implementation and Compute

All models were implemented in Python using PyTorch 2.10.0 and PyWavelets 1.8.0 for DWT layers, the DWT layers are wrapped so that gradients backpropagate through the linear transforms, keeping the full pipeline end-to-end trainable. Training was conducted on a single NVIDIA RTX-class GPU. Each run logs the model checkpoint with the best validation loss, along with seeds, hyperparameters, and metric traces; scripts to reproduce the experiments (data preparation, noise simulation, training, and analysis) are organized so that any configuration can be launched with a single command. To avoid confounding, we fixed the image normalization and augmentation across all methods and verified that the parameter counts are matched between max- and wavelet-pooled variants (as shown in Figure 1). We also measured single-image inference time on the same hardware in evaluation mode.

### 3.12. Summary of Experimental Grid

Putting the pieces together, our experiments cover:

- Pooling: Avg, Max, Sym2 (w/ and w/o preprocessing), Sym4 (w/preprocessing), Sym6 (w/preprocessing).
- Noise: 0%, 10%, 20% speckle.
- Depth:  B=3 and B=4.
- Batch size: 8, 16, 32.
- Baselines: VMC-NET (max pooling), VGG-style transfer.
- Repeats: 5 seeds per configuration.

This yields the 108 training/evaluation runs reported in Table 2, plus baseline comparisons and interpretability analyses. The design isolates the contribution of pooling (and optional preprocessing) while keeping training budget, optimizer, and data protocol constant, so differences can be attributed to the architectural choices we study.

Our choices aim to keep performance stable when ultrasound images are degraded by speckle. Multiplicative noise simulation mirrors coherent imaging physics in a simple, reproducible way. Wavelet-domain preprocessing attenuates high-frequency speckle while preserving edges, and wavelet pooling reduces resolution after a multiscale decomposition, retaining structure that max/average pooling may discard. Lightweight convolutional blocks with batch normalization support stable optimization; smaller batches add helpful stochasticity for heterogeneous medical data. Finally, non-parametric statistics (Friedman, Wilcoxon; DeLong for AUC) move comparisons from point estimates to tested differences, aligning the evaluation with our robustness objective.

**Table 2:** Classification Performance of CNN with Various Pooling Methods on Ultrasound Images Under Different Noise Levels.

Pooling Condition	Speckle Noise Level	Accuracy	Precision	Recall	F1-Score	AUC	Specificity
Avg Pooling	0%	77.61	77.70	77.61	77.59	87.04	74.72
Max Pooling	0%	84.04	86.27	84.04	83.79	93.63	96.45
Wavelet-Sym2 (no preprocessing)	0%	89.14	89.33	89.14	89.12	97.72	92.68
Wavelet-Sym2 (preprocessed)	0%	93.90	93.95	93.90	93.90	98.89	95.57
Wavelet-Sym4 (preprocessed)	0%	92.35	92.53	92.35	92.34	97.69	89.14
Wavelet-Sym6 (preprocessed)	0%	92.02	92.05	92.02	92.02	97.76	90.69
Avg Pooling	10%	80.16	80.16	80.16	80.16	87.87	80.04
Max Pooling	10%	84.37	84.38	84.37	84.37	91.81	85.37
Wavelet-Sym2 (no preprocessing)	10%	92.02	92.12	92.02	92.01	97.95	94.46
Wavelet-Sym2 (preprocessed)	10%	90.69	90.92	90.69	90.67	97.29	86.92
Wavelet-Sym4 (preprocessed)	10%	93.02	93.19	93.02	93.01	98.08	89.80
Wavelet-Sym6 (preprocessed)	10%	92.13	92.14	92.13	92.13	97.75	92.90
Avg Pooling	20%	78.94	79.17	78.94	78.89	86.35	83.37
Max Pooling	20%	82.48	82.51	82.48	82.48	90.26	84.04
Wavelet-Sym2 (no preprocessing)	20%	89.80	89.98	89.80	89.79	97.68	93.13
Wavelet-Sym2 (preprocessed)	20%	92.24	92.29	92.24	92.24	98.49	94.01
Wavelet-Sym4 (preprocessed)	20%	90.69	90.77	90.69	90.68	96.86	92.90
Wavelet-Sym6 (preprocessed)	20%	91.02	91.02	91.02	91.02	97.33	91.13

## 4. Results and Discussion

This section presents a comprehensive analysis of the experimental results, which collectively support the efficacy and robustness of the proposed wavelet-enhanced CNN architecture within the source-domain setting and under the controlled noise conditions examined in this study. The evaluation spans 108 experimental configurations involving different pooling methods, preprocessing strategies, network depths, batch sizes, and noise levels (0%, 10%, 20%). The results are discussed in terms of classification metrics, architectural efficiency, statistical significance, ablation outcomes, and interpretability through visualizations.

### 4.1. Performance Trends Across Pooling Strategies and Noise Levels

The comparative performance across pooling strategies clearly illustrates the consistent superiority of wavelet-based approaches, particularly those combined with preprocessing, over traditional max and average pooling methods. As shown in Table 2, under clean imaging conditions (0% speckle noise), the model employing Wavelet-Sym2 pooling with preprocessing achieved the best overall performance: 93.90% accuracy, 93.95% precision, 93.90% recall, 93.90% F1-score, 98.89% AUC, and 95.57% specificity. This suggests not only high discriminative power but also strong reliability in reducing false positives. In contrast, the best-performing conventional method, Max Pooling, reached only 84.04% accuracy and 93.63% AUC, nearly 10 points lower than the Sym2 configuration. Meanwhile, Average Pooling performed the worst, with only 77.61% accuracy and 74.72% specificity, highlighting its inadequacy in capturing subtle lesion features even under ideal conditions. Notably, Wavelet-Sym2 without preprocessing still achieved a strong 89.14% accuracy and 97.72% AUC, showing that wavelet pooling alone contributes significantly to effective feature preservation.

As noise levels increased to 10%, the performance gaps widened further, emphasizing the robustness of the proposed wavelet-based models. In this setting, Wavelet-Sym4 with preprocessing outperformed all configurations with 93.02% accuracy, 93.19% precision, 93.01% F1-score, and 98.08% AUC. Meanwhile, Wavelet-Sym2 (both with and without preprocessing) maintained strong performance (up to 92.02% accuracy, 97.95% AUC). On the other hand, traditional pooling methods showed greater sensitivity to noise: Max Pooling dropped to 84.37% accuracy, while Average Pooling fell to 80.16%, with specificity dropping to 85.37% and 80.04%, respectively. These results reinforce the advantage of wavelet pooling in preserving discriminative features under degradation, especially when combined with preprocessing techniques that suppress noise while maintaining structural detail. This dual-layer robustness, combining input-level denoising and architecture-level pooling, proves particularly effective for ultrasound-based malignancy classification.

At the highest tested noise level of 20% speckle, the trends held steady. Wavelet-Sym2 with preprocessing again led all configurations, yielding 92.24% accuracy, 92.29% precision, 92.24% F1-score, and a remarkable 98.49% AUC, along with 94.01% specificity. Sym4 and Sym6 with preprocessing also maintained robust performance at 90.69% and 91.02% accuracy, respectively. In stark contrast, conventional pooling suffered substantial degradation: Average Pooling fell to 78.94% accuracy and 86.35% AUC, while Max Pooling dropped to 82.48% accuracy and 90.26% AUC. These results illustrate the resilience of wavelet-enhanced architectures under the controlled simulated speckle conditions examined in this study, where image quality was deliberately degraded to test robustness. The consistently strong performance of wavelet-preprocessed configurations across all noise levels supports the practical value of this approach within the source-domain experimental setting, although the later external validation results indicate that broader cross-dataset generalization remains limited.

At the same time, these internal gains should be interpreted with some caution because the source-domain partitioning was performed at the image level rather than the patient level.

### 4.2. Baseline Comparison and Efficiency Trade-Off

To further contextualize the proposed model’s effectiveness, Table 3 compares its performance to a lightweight benchmark model, VMC-NET, under clean conditions. The proposed CNN using Wavelet-Sym2 pooling (preprocessed) achieved 93.90% accuracy, 93.90% F1-score, and 98.89% AUC, clearly outperforming VMC-NET, which recorded 92.90% accuracy, 92.90% F1-score, and 98.43% AUC. Although VMC-NET is significantly lighter (with 155,986 parameters vs. 565,658 in the proposed model), and marginally faster in inference (2.15 ms vs. 3.32 ms), the performance trade-off is non-trivial. The proposed architecture maintains reasonable efficiency while offering a notably more robust and reliable classification, which is especially important in clinical contexts where false negatives can have severe consequences. Therefore, while VMC-NET may be attractive for extreme edge scenarios, the proposed model shows a better balance between accuracy and computational practicality within the internal experimental setting considered in this study.

### 4.3. Statistical Validation of Pooling Performance

The statistical rigor of the observed results was evaluated using both Friedman and Wilcoxon signed-rank tests, as summarized in Table 4. The Friedman test applied under 0% noise revealed a statistically significant difference among pooling strategies with a *p*-value of 0.0002, far below the 0.05 threshold. The ranking order was as follows: Sym2 (preprocessed) > Sym4 > Sym6 > Sym2 (no preprocessing) > Max > Avg, confirming the superiority of wavelet-enhanced pooling, especially when combined with input-level denoising. To further validate pairwise differences under noisy conditions, the Wilcoxon test was applied. At 10% noise, Sym4 vs. Average pooling yielded a *p*-value of 0.0312, while at 20% noise, Sym2 (preprocessed) vs. Max pooling also resulted in a *p*-value of 0.0312. These findings underscore that the observed advantages are not only quantitatively measurable but also statistically robust within the present experimental setting, supporting further investigation of wavelet-based mechanisms for noise-stressed ultrasound classification.

### 4.4. Ablation Study: Depth and Batch Size Sensitivity

Table 5 presents an ablation study analyzing the effect of architectural depth and batch size on classification accuracy using the Wavelet-Sym2 pooling configuration. The 3-block architecture with batch size 8 consistently achieved the highest accuracy across all noise levels: 93.90% at 0%, 90.69% at 10%, and a notably strong 92.24% at 20% noise. This indicates that smaller batch sizes allow for better gradient stability and generalization, particularly under noisy data conditions. As batch size increased to 16 and 32, performance dropped progressively. For example, moving from batch size 8 to 32 led to a decrease from 90.69% to 83.92% at 0% noise and from 92.24% to 86.81% at 20% noise. This suggests that large batches may oversimplify the optimization landscape, especially in the presence of complex image noise.

Interestingly, the 4-block architecture did not consistently outperform its shallower counterpart. While batch size 16 provided competitive performance (e.g., 90.13% at 0% and 90.02% at 20% noise), the configuration struggled under batch sizes 8 and 32. Notably, the 4-block network with batch size 8 recorded only 83.81% accuracy at 10% noise, while with batch size 32 it dropped to 84.04% under 20% noise. These results suggest that simply increasing depth is not inherently beneficial and may lead to overfitting or instability without proper regularization. Overall, the findings support a design philosophy favoring leaner, more stable architectures trained with smaller batch sizes when robustness to controlled simulated degradation is an important objective.

Accordingly, the ablation trends reported here are best understood as stable patterns within the present source-domain experiment, rather than as direct estimates of patient-level clinical generalization.

**Table 5:** Effect of Batch Size on Classification Accuracy Using VGG-16 (5 Blocks) Under Speckle Noise.

No. of Blocks	Batch Size	Accuracy (0% Speckle Noise)	Accuracy (10% Speckle Noise)	Accuracy (20% Speckle Noise)
3	8	93.90	90.69	92.24
3	16	86.47	90.13	88.58
3	32	83.92	89.36	86.81
4	8	86.25	83.81	90.58
4	16	90.13	90.02	90.02
4	32	86.70	85.70	84.04

Table 5 evaluates VGG-16 (5 blocks, 14.8 M parameters) as a baseline deep architecture across batch sizes (8, 16, 32) and speckle noise levels (0%, 10%, 20%). Despite achieving 87.47% accuracy under clean conditions, VGG-16 exhibited significant performance degradation under noise, dropping to approximately 70% at 10% noise and 64–69% at 20% noise. This substantial accuracy decline (nearly 20 percentage points) demonstrates that deeper architectures with more parameters do not inherently provide noise robustness, reinforcing the advantage of our proposed wavelet-enhanced approach which maintains higher accuracy across all noise conditions with considerably fewer parameters.

Table 6 compares Max pooling (representing traditional pooling methods, as it outperformed Average pooling in previous experiments) against the proposed Wavelet-Sym2 pooling under Rayleigh noise conditions. The evaluation spans varying network depths (3 and 4 blocks), batch sizes (8, 16, 32), and Rayleigh noise levels (0%, 10%, 20%) to assess robustness against this distinct noise distribution.

As an additional exploratory robustness check, we also compared Max pooling and Wavelet-Sym2 pooling under a Rayleigh-type noise setting. This analysis was more limited than the main speckle-noise experiments and is therefore presented only as supplementary comparative evidence rather than as a central component of the study. As shown in Table 7, Wavelet-Sym2 generally remained competitive under this alternative noise model, although both methods showed noticeable degradation at higher noise levels.

### 4.5. Visual Interpretability and Robust Feature Localization

Beyond quantitative metrics, visual evidence of the model’s interpretability was obtained through architectural schematics and attention visualization. Figure 1 compares the internal layer configurations of three CNN variants. The average pooling model is notably smaller (61,362 parameters) but also the least performant. Both the max pooling and wavelet pooling variants maintain identical parameter sizes (565,658), ensuring that observed performance gains are due to the pooling mechanism rather than model complexity.

**Figure 3:**
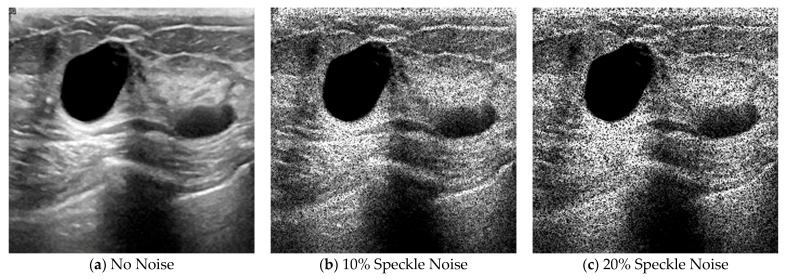
Examples of Ultrasound Images with Simulated Speckle Noise at Varying Levels. (**a**) Original image with no noise, (**b**) image with 10% simulated speckle noise (σ = 0.1), and (**c**) image with 20% simulated speckle noise (σ = 0.2). All images are resized to 224 × 224 pixels.

To further validate robustness, Figure 3 displays the same ultrasound image with 0%, 10%, and 20% speckle noise. As noise increases, lesion visibility and texture sharply deteriorate, emphasizing the need for architectures capable of extracting stable features under noisy conditions. Complementing this, Figure 2 uses Grad-CAM heatmaps to show attention differences between pooling strategies. Models with average pooling produce scattered attention, while max pooling achieves better focus. However, only the preprocessed wavelet model achieves tightly localized lesion focus, supporting its qualitative interpretability and suggesting better preservation of lesion-relevant features.

**Figure 4:**
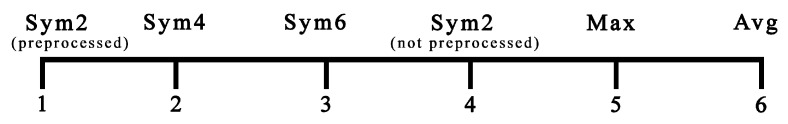
Critical Difference Diagram Showing Statistical Ranking of Pooling Methods Based on Friedman Test Across All Experiments.

Further validating these differences, Figure 4 presents a Critical Difference (CD) diagram, ranking all pooling methods based on performance. Sym2 (preprocessed) ranks highest, followed by Sym4 and Sym6. The conventional methods, Max and Average pooling, occupy the lowest ranks, with the statistical gap indicating significant performance divergence. This confirms that the proposed strategy offers consistent and meaningful gains across experiments.

**Figure 5:**
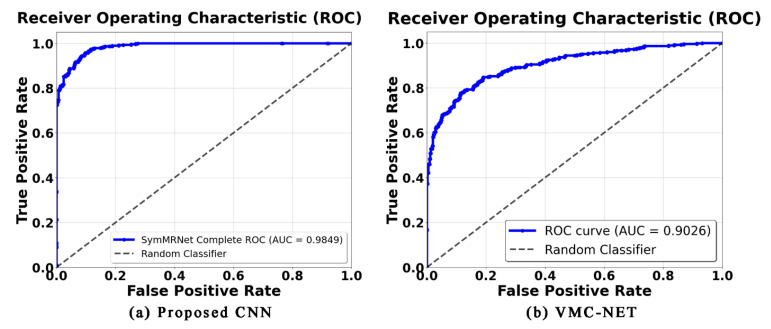
ROC Curves for Top Performing Configurations Under 20% Speckle Noise. (**a**) ROC curve for the proposed CNN (Sym2 + preprocessing), and (**b**) ROC curve for VMC-NET (Maxpooling baseline).

Finally, Figure 5 presents ROC curves under the most challenging setting (20% noise). The steeper, earlier-rising curve of the proposed model indicates stronger discrimination under this internal 20% speckle condition. This visual evidence complements the statistical and tabular findings, supporting wavelet pooling as a useful architectural direction for improving in-domain performance under controlled perturbation.

Taken together, our experiments show that wavelet-based pooling delivers consistent advantages over conventional down-sampling across clean and noisy ultrasound, with the largest margins appearing as noise increases. Under 0% noise, Wavelet-Sym2 with preprocessing achieved 93.90% accuracy, 93.95% precision, 93.90% recall/F1, 98.89% AUC, and 95.57% specificity, outperforming Max Pooling (84.04% accuracy) and Average Pooling (77.61% accuracy, 74.72% specificity); importantly, even Sym2 without preprocessing reached 89.14% accuracy and 97.72% AUC, indicating that multiscale pooling alone helps preserve structure. At 10% noise, Sym4 with preprocessing led with 93.02% accuracy, 93.19% precision, 93.01% F1, and 98.08% AUC, while Max and Average dropped to 84.37% and 80.16% accuracy (specificity 85.37% and 80.04%), respectively. At 20% noise, Sym2 with preprocessing again topped the table (92.24% accuracy, 92.29% precision, 98.49% AUC, 94.01% specificity), whereas Average and Max fell to 78.94% (AUC 86.35%) and 82.48% (AUC 90.26%). These gains are statistically supported: the Friedman test at 0% noise yielded *p* = 0.0002, ranking Sym2 (preprocessed) > Sym4 > Sym6 > Sym2 (no prep) > Max > Avg, and Wilcoxon tests at 10% and 20% noise confirmed Sym4 vs. Avg and Sym2 (prep) vs. Max with *p* = 0.0312. Qualitatively, Grad-CAM maps show tighter, lesion-centric focus for wavelet configurations, and the CD diagram visualizes their rank separation. Practically, our proposed CNN (Sym2, with preprocessing) pairs higher accuracy (93.90% vs. 92.90%) and AUC (98.89% vs. 98.43%) with acceptable cost (565,658 vs. 155,986 parameters; 3.32 ms vs. 2.15 ms inference) relative to VMC-NET, suggesting a favorable performance–efficiency balance within the present internal evaluation setting. The ablation indicates that a 3-block network with batch size 8 is most stable (e.g., 90.69% at 0% and 10% noise; 92.24% at 20% noise), whereas deeper/larger-batch settings can underperform (e.g., 3-block bs = 32 to 83.92% at 0% noise; 4-block bs = 32 to 84.04% at 20% noise). Limitations include the use of simulated speckle rather than probe-specific noise profiles and evaluation on a single dataset; future work should test multi-site data, incorporate device/domain adaptation, calibrate operating points (e.g., DeLong-tested AUC differences and clinically tuned sensitivity), and explore even lighter wavelet layers for edge devices. Overall, the data support wavelet pooling, especially with preprocessing, as a simple, effective modification that improves robustness without prohibitive computational cost.

### 4.6. External Validation and Cross-Dataset Generalization

For external validation, the best internal model checkpoint, selected according to the internal validation procedure, was directly applied to the independent BrEaST-Lesions-USG dataset without retraining or parameter adaptation. The purpose of this experiment was to examine out-of-domain transfer under a strict setting. The same inference pipeline was retained, and the reported threshold corresponds to the decision setting used during this external evaluation. Because no recalibration or domain adaptation was applied, the resulting performance can be interpreted as a direct measure of raw cross-dataset generalization ability. The threshold was inherited from the internal validation-based model selection stage and was not re-optimized on the external dataset. To assess whether the observed internal robustness could transfer beyond the source-domain setting, the best-performing configuration from the internal experiments—namely, Sym2 + preprocessing—was evaluated on the independent BrEaST-Lesions-USG dataset without retraining. In contrast to the strong internal results, the external evaluation revealed substantial performance deterioration (Table 8). Accuracy decreased from 93.90% on the internal test set to 53.97% on the external dataset, while ROC AUC dropped from 98.89% to 0.4713. Sensitivity was especially low at 39.80%, meaning that only 39 of 98 malignant lesions were correctly identified, whereas 59 malignant cases were incorrectly classified as benign. Specificity reached 62.99%, with 97 of 154 benign cases correctly recognized. These results indicate that the proposed architecture, although effective under internal clean and simulated noisy conditions, remained highly vulnerable to cross-dataset distributional differences.

This marked degradation suggests that robustness to controlled synthetic speckle perturbation should not be interpreted as robustness to real-world inter-dataset variation. In breast ultrasound imaging, domain shift may arise from differences in scanner manufacturers, acquisition protocols, image post-processing pipelines, lesion composition, annotation style, and patient population characteristics. Such inter-institutional variability has been empirically demonstrated in multicenter validation studies, where model performance was found to differ substantially across hospital types and patient populations [55]. The present findings therefore highlight an important distinction between source-domain robustness and true external generalization. In particular, the low sensitivity observed here represents a clinically critical failure mode, since missed malignant lesions are more concerning than increased false positives in many diagnostic settings. Accordingly, the current results should be interpreted as evidence that wavelet-based pooling improves in-domain stability and feature preservation, but is not by itself sufficient to guarantee reliable performance across independent clinical datasets.

The extremely low decision threshold used during external inference (0.0007) also indicates that the model outputs became poorly calibrated under domain shift. To avoid overstating the practical readiness of the proposed approach, we emphasize that the external validation findings substantially narrow the scope of the manuscript’s claims. The main practical conclusion of this work is therefore not that cross-institutional robustness has been solved, but rather that wavelet pooling constitutes a useful architectural direction whose benefits are currently demonstrated within the source-domain setting and under controlled perturbation experiments.

Several factors likely contributed to this domain gap. First, the two datasets were acquired with different ultrasound systems and scan settings: the Kaggle source was compiled from multiple contributors with unstated device parameters, producing systematically different gain, dynamic range, and speckle statistics. Second, annotation conventions differ, as the external cohort uses radiologist-validated BI-RADS categories while the source relies on compiled labels. Third, class prevalence differs, which shifts the operating point of the learned decision boundary. Fourth, our controlled speckle augmentation during training modeled multiplicative Gaussian noise only, which does not capture scanner-specific post-processing such as harmonic imaging, spatial compounding, or proprietary speckle reduction filters present in clinical systems. Potential mitigation strategies that we identify as necessary future work include (i) unsupervised domain adaptation approaches such as feature-level CORAL alignment or adversarial domain-invariant training, (ii) test-time batch-normalization statistics adaptation, (iii) intensity normalization and histogram matching between source and target cohorts prior to inference, (iv) retraining on a multi-institutional pool to expose the network to broader acquisition variability, and (v) decision threshold recalibration against a small labeled target subset. These are beyond the scope of the present architectural study, but are flagged as priorities for translational deployment.

The confusion matrix (Table 8) further illustrates the classification challenges, with the model correctly identifying only 39 of 98 malignant cases (39.80% sensitivity) while misclassifying 59 malignant lesions as benign a critical failure mode with severe clinical implications. Similarly, among 154 benign cases, 97 were correctly classified (62.99% specificity), while 57 were incorrectly identified as malignant.

Table 9 shows the confusion matrix for external validation, highlighting severe generalization challenges. The model achieved only 39.80% sensitivity (39/98 malignant cases correctly identified), with 59 malignant lesions misclassified as benign a critical clinical failure. Specificity was 62.99% (97/154), with 57 benign cases incorrectly flagged as malignant. The extremely low classification threshold (0.0007) indicates poor model confidence on external data, confirming substantial domain shift between training and validation datasets.

Table 9 further confirms that the principal failure mode was the misclassification of malignant lesions as benign, which is the most concerning type of error from the clinical viewpoint.

The present study does not include calibration analysis (e.g., Brier score, Expected Calibration Error, or reliability diagrams) nor a systematic quantitative evaluation of Grad-CAM localization against annotated lesion boundaries. These constitute recognized best practices for clinically translatable AI systems and represent important limitations of the current work. The primary contribution of this study is an architectural comparison of pooling strategies under speckle noise, calibration assessment and localization fidelity analysis are planned as explicit objectives in subsequent prospective validation work prior to any clinical deployment consideration.

This study has several limitations that should be acknowledged. First, the robustness experiments were mainly conducted under simulated speckle perturbation, which, although useful for controlled stress testing, cannot fully represent the complexity of real-world scanner-specific noise and post-processing effects in clinical ultrasound imaging. Second, the main model development process was performed within a single source-domain dataset, and the external validation results showed that strong internal performance did not translate to satisfactory cross-dataset discrimination. Third, the observed performance degradation on the independent dataset indicates that the proposed architectural improvement alone is not sufficient to overcome domain shift. For this reason, the present work should be interpreted primarily as an architectural proof-of-concept for improving in-domain resilience rather than as evidence of immediate deployment readiness.

## 5. Conclusions

This study introduced a wavelet-enhanced convolutional neural network architecture for breast cancer classification in ultrasound imaging, targeting robustness under varying levels of speckle noise. By systematically comparing six pooling strategies, including average, max, and three Symlet-based wavelet pooling configurations (with and without preprocessing), across multiple network depths, batch sizes, and controlled noise levels (0%, 10%, and 20%), the results consistently showed that wavelet pooling, particularly when combined with wavelet-domain input preprocessing, outperforms conventional pooling in both accuracy and stability. The best-performing model (Sym2 + preprocessing) achieved 93.90% accuracy and 98.89% AUC under clean conditions and maintained strong performance at higher noise levels (e.g., 92.24% accuracy and 98.49% AUC at 20% noise). These improvements were statistically validated using Friedman and Wilcoxon tests and further supported by critical difference diagrams and Grad-CAM heatmaps, which confirmed better lesion localization in the wavelet-based models. Compared to a well-known lightweight CNN (VMC-NET), our architecture offered better classification performance with only a modest increase in parameter count and inference time. The architecture ablation study highlighted that a 3-block model with a batch size of 8 offered the best robustness–efficiency trade-off, while larger batch sizes or deeper networks occasionally underperformed.

In conclusion, this study shows that Symlet-based wavelet pooling, particularly when combined with wavelet-domain preprocessing, can provide clear benefits for breast ultrasound classification within the source-domain setting. Across clean and controlled speckle-corrupted conditions, the proposed models achieved stronger accuracy, AUC, and stability than conventional pooling-based alternatives, while also showing more lesion-focused activation behavior in Grad-CAM analysis. At the same time, the external validation results revealed a substantial loss of performance, with accuracy decreasing to 53.97%, ROC AUC to 0.4713, and sensitivity to 39.80% on an independent dataset. These findings indicate that the present method should be understood as an effective in-domain robustness enhancement rather than as a fully generalizable clinical solution. Therefore, the main value of this work lies in demonstrating that wavelet-based pooling is a simple and computationally feasible architectural direction for improving resilience to controlled noise, while also making it clear that broader clinical applicability will require further advances in cross-dataset adaptation, calibration, and generalization.

## Figures and Tables

**Table 1:** Dataset Characteristics.

Dataset	Source	Total Samples	Benign	Malignant	Purpose
Ultrasound Breast Images for Breast Cancer	Friedman	9016	4574	4442	Training, validation, internal testing
BrEaST-Lesions-USG	TCIA	252	154	98	External validation

**Table 3:** Performance Comparison Between the Proposed CNN and Benchmark Models (VGGNet, etc.).

Model	Pooling Method	Speckle Noise Level	Accuracy	F1-Score	AUC	Params	Inference Time (ms)
Proposed CNN	Wavelet-Sym2 (preprocessed)	0%	93.90	93.90	98.89	565,658	3.32
VMC-NET	Max Pooling	0%	92.90	92.90	98.43	155,986	2.15
VGG-16	Average Pooling	0%	87.47	87.47	95.06	14,813,570	3.96

**Table 4:** Friedman and Wilcoxon Tests Across Pooling Methods for Different Noise Conditions.

Speckle Noise Level	Test Type	Ranking Order (Best to Worst)	*p*-Value	Significant?
0%	Friedman	Sym2 (preprocessed) > Sym4 > Sym6 > Sym2 (no preprocessing) > Max > Avg	0.0002	Yes
10%	Wilcoxon (Sym4 vs. Avg)	-	0.0312	Yes
20%	Wilcoxon (Sym2 vs. Max)	-	0.0312	Yes

**Table 6:** Effect of Network Depth and Batch Size on Classification Accuracy Using VGG-16 (5 Blocks).

Batch Size	Accuracy (0% Noise)	Accuracy (10% Speckle Noise)	Accuracy (20% Speckle Noise)
8	87.14	69.51	65.96
16	87.47	70.07	69.40
32	84.48	68.29	63.86

**Table 7:** Effect of Network Depth and Batch Size on Classification Accuracy Using Wavelet-Sym2 Pooling. Bold values indicate the best performance per noise condition within each configuration.

No. of Blocks	Batch Size	Pooling	Accuracy (0% Rayleigh Noise)	Accuracy (10% Rayleigh Noise)	Accuracy (20% Rayleigh Noise)
3	8	Max	84.04	78.05	70.07
		**Sym2**	**93.90**	80.37	**78.82**
	16	Max	84.92	75.28	56.10
		**Sym2**	86.47	**83.70**	58.20
	32	Max	80.93	81.04	60.75
		Sym2	83.92	82.15	67.63
4	8	Max	93.35	78.60	57.87
		Sym2	86.25	83.48	65.96
	16	Max	92.90	80.93	68.51
		Sym2	90.13	83.48	67.07
	32	Max	91.02	81.37	54.77
		Sym2	86.70	80.16	56.43

**Table 8:** External Validation Performance on Brast-Lesions-USG Dataset.

Metric	Value
Accuracy	53.97%
Sensitivity (Recall)	39.80%
Specificity	62.99%
F1-Score	0.4021
ROC AUC	0.4713

**Table 9:** Confusion Matrix for External Validation (Threshold = 0.0007).

	Predicted Benign	Predicted Malignant
Actual Benign	97	57
Actual Malignant	59	39

## Data Availability

The original data presented in this study are derived from publicly available resources. The Ultrasound Breast Images for Breast Cancer dataset is publicly available on Kaggle at https://www.kaggle.com/datasets/vuppalaadithyasairam/ultrasound-breast-images-for-breast-cancer (accessed on 24 August 2025). The BrEaST-Lesions-USG dataset used for external validation is publicly available at https://doi.org/10.7937/9WKK-Q141 (accessed on 24 August 2025).
